# Pathogens and Politics: Further Evidence That Parasite Prevalence Predicts Authoritarianism

**DOI:** 10.1371/journal.pone.0062275

**Published:** 2013-05-01

**Authors:** Damian R. Murray, Mark Schaller, Peter Suedfeld

**Affiliations:** Department of Psychology, University of British Columbia, Vancouver, British Columbia, Canada; University of California Los Angeles, United States of America

## Abstract

According to a "parasite stress" hypothesis, authoritarian governments are more likely to emerge in regions characterized by a high prevalence of disease-causing pathogens. Recent cross-national evidence is consistent with this hypothesis, but there are inferential limitations associated with that evidence. We report two studies that address some of these limitations, and provide further tests of the hypothesis. Study 1 revealed that parasite prevalence strongly predicted cross-national differences on measures assessing individuals' authoritarian personalities, and this effect statistically mediated the relationship between parasite prevalence and authoritarian governance. The mediation result is inconsistent with an alternative explanation for previous findings. To address further limitations associated with cross-national comparisons, Study 2 tested the parasite stress hypothesis on a sample of traditional small-scale societies (the Standard Cross-Cultural Sample). Results revealed that parasite prevalence predicted measures of authoritarian governance, and did so even when statistically controlling for other threats to human welfare. (One additional threat—famine—also uniquely predicted authoritarianism.) Together, these results further substantiate the parasite stress hypothesis of authoritarianism, and suggest that societal differences in authoritarian governance result, in part, from cultural differences in individuals' authoritarian personalities.

## Introduction

Systems of governance differ widely, and one important dimension on which they vary is authoritarianism. In contrast to liberal democratic forms of governance (characterized by popular participation in the political process, and by protection of individual civil rights and ideological freedoms), authoritarian governance is defined by highly concentrated power structures that repress dissent and emphasize submission to authority, social conformity, and hostility towards outgroups [1], [2]. Why is governance in some states and societies more authoritarian than in others? Economic variables—including the overall availability of economic resources and the manner in which those resources are distributed—provide partial answers to that question [3], [4], [5], [6]. Ecological variables may play a role as well. Recently, it has been suggested that societal variability in authoritarian governance may result, in part, from variability in the prevalence of disease-causing parasites [7]. (In this context, “parasite” is used to refer broadly to any pathogenic organism, including bacteria and viruses as well as helminths). Although results from several initial studies support this “parasite stress” hypothesis of authoritarian governance [7], [8], alternative explanations for those results remain unaddressed. Here, we report results from two additional investigations designed to test the parasite stress hypothesis and to address inferential limitations of previous studies.

Why might there be a causal link between the prevalence of infectious diseases in the local ecology and an authoritarian system of governance? The hypothesis follows from an analysis of several defining characteristics of authoritarian political systems (such as institutionalized emphasis on social conformity, intolerance of dissent, and ethnocentrism) that may have implications for the spread of infectious disease. Because many disease-causing parasites are invisible, and their actions mysterious, disease control has historically depended substantially on adherence to ritualized behavioral practices that reduced infection risk [9]. Individuals who openly dissented from, or simply failed to conform to, these behavioral traditions therefore posed a health threat to self and others. Thus, while there can be societal costs associated with any collective behavioral tendency toward obedience and conformity (e.g., inhibition of technological innovation), there can be disease-specific benefits too (presuming that a greater proportion of these behavioral traditions serve to mitigate, rather than propagate, the spread of disease). These benefits would have been greater (and more likely to outweigh the costs) under circumstances in which disease-causing parasites placed greater stress on human welfare—circumstances in which those parasites were especially virulent and/or prevalent.

This logical analysis has implications for predictable variation in individuals' attitudes and values, and for worldwide societal differences too. At a psychological level of analysis, empirical evidence reveals that the subjective perception of infection risk causes individuals to be more conformist, to prefer conformity and obedience in others, to respond more negatively toward others who fail to conform, and to endorse more conservative socio-political attitudes [10], [11], [12], [13], [14]. At a societal level of analysis, empirical evidence reveals that in countries and cultures characterized by historically higher prevalence of parasitic diseases, people are less individualistic, exhibit lower levels of dispositional openness to new things, are more likely to conform to majority opinion, and more strongly endorse "binding" moral values that emphasize group loyalty, obedience, and respect for authority [15], [16], [17], [18], [19].

In addition to their intolerance of nonconformity, authoritarian political systems are also characterized by nepotism and ethnocentrism [20]. These behavioral tendencies too have been empirically linked to the threat of disease. At a psychological level of analysis, individuals who are—or who merely perceive themselves to be—more vulnerable to infection tend to endorse more xenophobic and ethnocentric attitudes [21], [22], [23]. At a societal level of analysis, countries characterized by higher prevalence of parasitic diseases are also characterized by stronger family ties, increased frequency of intrastate ethnic conflict, and several indicators of increased ethnocentrism [24], [25], [26], although the interpretation of some these results remains a matter of some disagreement [27], [28].

To the extent that institutionalized forms of governance reflect the attitudes and values of the individuals who populate the local ecology, these lines of research have implications for predicting worldwide variability in authoritarian governance: In places where parasitic diseases have posed greater stress on human health and welfare, authoritarian forms of governance may be especially likely to emerge and to persist over time.

Thornhill and colleagues [7] empirically tested this parasite stress hypothesis, using modern geopolitical entities (e.g., countries) as units of analysis. The hypothesis was tested on four different measures of democratization and/or authoritarianism, using a parasite stress measure derived from a modern epidemiological database. Consistent evidence was observed across all measures: Higher levels of parasite stress were associated with less democratic, more highly authoritarian political systems (*N*’s >192, absolute *r*’s >.45, *p*’s<.001). These relationships remained statistically significant when statistically controlling for measures of economic development and economic inequality (as assessed by a country’s GDP per capita and GINI coefficient respectively). Additional analyses revealed that country-level differences in authoritarian governance were even more strongly predicted by a measure of *historical* (rather than modern-day) parasite prevalence [29]—a finding consistent with the hypothesis that authoritarian governance is a consequence (rather than a cause) of parasite stress.

However, nontrivial inferential issues arise from the use of contemporary nation-states as units of analysis [30]. Until these issues are addressed empirically, it is difficult to draw confident conclusions about the relationship between parasite stress and authoritarian governance.

One issue pertains to the history of European colonization, and its consequences. When countries colonize other geographic regions, they often impose their own political and economic institutions onto those regions; those institutions may persist even after those regions attain independence. It has been argued that ecological variables (such as the prevalence of infectious diseases) predict societal outcomes primarily because of their influence on particular patterns of colonial settlement, such that European colonial powers were more likely to establish long-lasting democratic political systems and economic institutions in regions characterized by lower incidences of infectious diseases [31], [32]. This represents a very different causal process than that implied by the parasite stress hypothesis.

We conducted two separate investigations, using two different empirical strategies, to address this inferential issue and thus to more rigorously test the hypothesized relation between parasite stress and authoritarian governance.

The first study revisits the country-level analyses reported previously [7]. The alternative explanation—differential colonial establishment of political institutions—is tested by examining relations not only between disease prevalence and state-level authoritarianism evident in government institutions, but also by examining the relation between those variables and authoritarian attitudes expressed by individuals who populate the country. The colonial-establishment-of-institutions explanation implies a direct causal influence of disease prevalence on state-level authoritarian governance, which may in turn have downstream consequences for individual-level authoritarian attitudes. Conversely, the parasite stress hypothesis implies a more direct causal influence of disease prevalence on individuals' authoritarian attitudes, which in turn would be expected to have a consequent influence on state-level systems of government. In statistical analytic terms, the alternative explanation implies an indirect relation between disease prevalence and individuals' authoritarian attitudes that is statistically mediated by authoritarian governance, whereas the parasite stress hypothesis implies an indirect relation between disease prevalence and authoritarian governance that is statistically mediated by individuals' authoritarian attitudes.

In addition to testing the alternative explanation, the results of this study also have implications for our understanding of individual-level authoritarian attitudes as they relate to societal outcomes. Research on “the authoritarian personality” of individuals indicates some relation between politically entrenched authoritarian systems of governance and individually expressed authoritarian personality traits (as such governments and individuals have in common their emphasis on adherence to conventional values, repression of dissent, and devotion to order and hierarchy [1], [2], [33]). But the direction of causality is unclear: To what extent does the correlation reflect the influence of government institutions on individuals' personalities, versus the influence of individuals' personalities on systems of governance? By introducing an additional variable into the analysis, and testing statistical mediation, our results may contribute toward some resolution to this question.

The second study further addresses alternative explanations based on European colonialism by testing the parasite stress hypothesis on a sample of more traditional societies documented within the Standard Cross-Cultural Sample [34]. The Standard Cross-Cultural Sample (SCCS) consists of 186 worldwide cultural populations, many of which are small-scale aboriginal societies. Cashdan and Steele [15] employed the SCCS dataset to test several other hypothesized consequences of disease prevalence that had previously been tested only with cross-national comparisons; their results provided important substantiation for the relationship between disease prevalence and collectivist values—especially those values pertaining to adherence to group norms. We employed the same strategy to provide an empirically complementary test of the hypothesis that ecological variation in disease prevalence predicts societal variation in authoritarian governance.

Drawing on an extensive ethnographic database, the cultures that comprise the SCCS are described by hundreds of numerically coded variables gathered by dozens of different ethnographers—including multiple variables pertaining to systems of governance [35]. These variables, in conjunction with two indicators of disease prevalence [15], [36], allow a statistically rigorous test of the relation between parasite stress and authoritarian governance in small-scale societies. Additional variables assess the prevalence of conceptually distinct threats to human welfare within these societies (e.g., famine, warfare). This allowed us to test the unique predictive effects of parasite stress, while statistically controlling for any effects associated with these other threats.

## Study 1

### Method

Analyses were conducted on 31 countries for which empirical data were available for the variables of primary conceptual interest: (a) authoritarian governance, (b) individual authoritarianism, and (c) historical prevalence of disease-causing parasites. The complete data for this study are available at http://www2.psych.ubc.ca/~schaller/datasets/MurraySchallerSuedfeld-Study1.sav; all statistical analyses were conducted using SPSS version 16.0.

#### Authoritarian Governance

We employed four variables that are either directly or inversely indicative of authoritarian governance; previous analyses reveal that all four variables are predicted by measures of parasite stress [7], [29].

Two measures were obtained from www.freedomhouse.org which, for all countries, provides numerical values indicating (1) governmental restrictions on individuals' political rights and (2) governmental restrictions on individuals' civil liberties. Both scores are represented on 7-point scales, with higher values indicating more severe governmental restrictions on individuals' rights and civil liberties. We employed scores pertaining to the year 2007.

A third measure (obtained from www.heritage.org) assesses the extent to which the law protects the rights of individuals to own and accrue private property. Country level scores were represented on a 100-point scale, with higher values representing greater legal protection for individuals' property rights (indicative of a lower level of authoritarian governance). We employed scores from the years 2004–2008.

The fourth measure was Vanhanen’s [6] index of democracy for the years 1999–2001. This index was derived from two components of a democratic governance system—competition and participation in the electoral process—which were weighted equally in computation of the overall democracy index. Higher values indicate higher levels of democratization (and lower levels of authoritarian governance) within each country.

#### Individual Authoritarianism

Adorno and colleagues [1] developed a questionnaire—the "F Scale"—to assess individual differences in traits and attitudes that define the authoritarian personality (e.g., conventionalism, authoritarian submission, authoritarian aggression, ethnocentrism). This scale has been validated in both Western and non-Western cultures [37]. Meloen [33] compiled results obtained from over 30,000 individuals worldwide who completed the F Scale, and reported mean standardized F Scale scores for individuals living within each of 31 countries. Meloen reported separate mean F Scale scores for student and non-student samples within each country. Although mean F Scale scores were different across these two types of samples (non-student means were generally higher), these two scores were almost perfectly correlated across the 31 countries (*r*>.99), and so virtually identical results emerge regardless of which set of scores is used as an indicator of individuals' authoritarian personality. In the analyses reported below, we employed mean F Scale scores obtained from non-student samples.

#### Prevalence of Disease-Causing Parasites

Parasite stress was assessed with a previously developed measure of historical pathogen prevalence [29]. This measure is based on the incidence rates of 9 different kinds of infectious diseases, as indicated in old medical atlases and other sources of historical information about disease prevalence in different geographical regions (the nine disease-causing pathogens that were coded were leishmanias, trypanosomes, leprosy, schistosomes, filariae, tuberculosis, malaria, dengue, and typhus). The resulting index provides, for each country, a numerical estimate of the relative overall historical prevalence of disease. (This measure is internally reliable; across a worldwide sample of 160 countries, the 9-item index has Cronbach's alpha  = .84).

#### Control Variables

Country-level variables other than disease prevalence may also predict authoritarianism, and it is important that these variables be accounted for statistically in order to test the unique predictive effects of disease prevalence. In our analyses, we included four such variables. (Given that these variables are ostensibly predictors—rather than consequences—of authoritarianism, we attempted to obtain measures that predated data collection on the primary authoritarianism measures, while still retaining accurate data for as many countries as possible.)

#### GDP per capita

Previous research indicates that authoritarian governance is associated with low levels of economic development [5]. As a measure of economic development, we used the World Bank's (www.data.worldbank.org) country-level scores of GDP per capita data for the year 1980.

#### Wealth Inequality

Inequitable distribution of wealth predicts variation in democratic versus authoritarian governance [6]. As a measure of wealth inequality we used GINI coefficients obtained from CIA World Factbook (www.cia.gov). (A GINI coefficient of zero indicates total wealth equality within a country; a coefficient of one indicates maximal inequality.) The dates of these GINI scores ranged from 1991–1996.

#### Education

Meloen [33] found that authoritarianism was inversely related to the mean level of education within a country. As a measure of this construct, we used the United Nations Education Index scores (obtained from www.hdr.undp.org) for the year 1990.

#### Life Expectancy Residual

In addition to the specific threat posed by infectious diseases, other threats to human welfare have also been found to be predictive of individuals' authoritarian personality and related attitudes [38], [39], [40], [41], [42]. We employed a method used in previous cross-national investigations [16] to create an index that indirectly assessed disease-*ir*relevant threats: We regressed average life expectancy (obtained for the year 1990, from www.hdr.undp.org) on the index of disease prevalence and saved the residuals. These life expectancy residuals represent variation in life expectancy that cannot be predicted by variation in disease prevalence, and thus is indicative of various other threats to human welfare.

### Results and Discussion

Preliminary analyses revealed that pathogen prevalence strongly predicted all four measures of authoritarian governance (*r*’s ranged from .47 to .67 in absolute value, all *p*’s<.01). These results replicate previous findings [7] on the smaller subset of countries included in our analyses.

Additional results (summarized in Table 1) revealed that individuals' authoritarianism scores were also strongly predicted by pathogen prevalence, *r* = .65 (*p*<.001). Individual authoritarianism was also predicted by GDP per capita, GINI, Education, and Life Expectancy Residual (*r*'s ranged from .36 to .68 in absolute value, all *p*'s<.05).

**Table 1:** Results from analyses on 31 countries (Study 1): Correlations between mean individual F-scale scores, historical pathogen prevalence, and other country-level variables.

	1	2	3	4	5
1. Mean F-Scale Scores	—				
2. Pathogen Prevalence	.65**	—			
3. Education Index	–.60**	–.73**	—		
4. GDP per Capita	–.68**	–.77**	.77**	—	
5. Wealth Inequality (GINI)	.51**	–.60**	–.42*	–.57**	—
6. Life Expectancy (Residual)	–.36*	.00	.41*	.38	–.26

Note: ** *p*<.01, * *p*<.05, N = 31.

In order to test unique effects on individuals' authoritarian scores, pathogen prevalence and the four control variables were entered simultaneously as predictors into a regression equation, with individual authoritarianism as the dependent variable. Results revealed that the predictive effects of the control variables were all statistically nonsignificant (absolute values of *β*’s<.32, *p*’s >.10), but that—despite relatively low statistical power—there was a significant unique effect of pathogen prevalence, *β* = .73 (*p* = .04). (The partial correlation coefficient between pathogen prevalence and authoritarian scores when controlling for these four control variables was *r* = .48, *p* = .04). This result is conceptually consistent with previous research linking pathogen prevalence uniquely to other conformist attitudes and personality traits 17,18.

The key question is whether the relation between pathogen prevalence and authoritarian governance is mediated by individual-level authoritarianism. To address this question, we employed a bootstrapping procedure [43]. We determined path coefficients by regression analyses, and determined indirect effects and their 95 percent confidence intervals based upon 10,000 nonparametric bootstrapped samples. Results of all 4 mediation tests indicated that the relation between pathogen prevalence and authoritarian governance was significantly mediated by individual authoritarianism, with none of the bootstrapped confidence intervals containing zero. The mediated effect accounted for 77% of the total effect of pathogen prevalence on political rights (Unstandardized regression coefficients [*B'*s] between pathogen prevalence and political rights  = .29/1.28 after/before mediation), for 63% of the total effect on civil liberties (*B*'s  = .50/1.38 after/before mediation), for 60% of the total effect on democracy (*B*'s  = –3.45/–8.60 after/before mediation, and for 37% of the effect on property rights (*B*'s  = –16.00/–25.56 after/before mediation). Of the 4 measures of authoritarian governance, only for the measure of property rights did the direct effect of pathogen prevalence remain significant when statistically controlling for individual authoritarianism (*β* = –.48, *p* = .02); for the remaining three measures of authoritarian governance, the direct effect of pathogen prevalence was reduced to nonsignificance (absolute values of *β*'s<.23, *p*'s >.20).

For the sake of comparison, we performed another set of bootstrapping analyses that tested an alternative mediational model that specified authoritarian governance as the mediator between pathogen prevalence and individual Authoritarianism. This alternative model was not as well supported by the data. Across the four analyses, the mediated effect accounted for between 31–48% of the total effect of pathogen prevalence on individual authoritarianism. The direct effect of pathogen prevalence on individual Authoritarianism remained significant in every analysis when controlling for state-level authoritarian governance (*p*’s ranged from .005 – .04).

These mediation results suggest that the ecological prevalence of infectious diseases predicts the individual authoritarian personalities of people living within that ecological region, and these individual-level dispositions in turn give rise to (and sustain) authoritarian systems of governance. These results are consistent with the logical implications of the parasite stress hypothesis, and are inconsistent with an alternative explanation suggesting that the correlation between disease prevalence and authoritarianism is based solely on colonial establishment of state-level institutions.

## Study 2

### Method

Analyses were conducted on 90 cultural populations described within the Standard Cross Cultural Sample (SCCS) [34], for which empirical data were available for the variables of primary conceptual interest: (a) authoritarian governance, and (b) historical prevalence of infectious disease. Complete data for this study are available at http://www2.psych.ubc.ca/~schaller/datasets/MurraySchallerSuedfeld-Study2.sav; all statistical analyses were conducted using SPSS version 16.0.

#### Authoritarian Governance

Drawing on ethnographic observations, Ross [35] coded 42 numerical variables assessing aspects of political life in 90 societies within the SCCS. Factor analytic results reported by Ross revealed that 12 of these variables loaded highly on a common underlying factor, which Ross called "concentration of political power"—a defining feature of authoritarian political systems. These variables are: Political role differentiation, Basis of local community leadership selection, Perceptions of political leaders’ power (as seen by society), Checks on leaders’ power, Removal of leaders who are incompetent or disliked, Leaders’ exercise of authority, Operation of decision making bodies, Extensiveness of adult participation in community decisions, Litigation/use of third parties for binding decisions, Formal sanctions and enforcement for community decisions, Prevalence of enforcement specialists (e.g. police, tax collectors), and Level of taxation paid to local community (SCCS variable numbers 756, 758, 759, 761, 762, 763, 764, 766, 772, 776, 777, 784). Due to society-specific missing (or insufficiently precise) data, *N*'s for these variables range from 77 to 90.

We employed all 12 of these variables as indicators of authoritarian governance. As originally coded, greater authoritarianism (i.e., greater concentration of political power) was indicated by lower numerical values on 11 of the 12 variables (all except SCCS variable #766, Extensiveness of adult participation in community decisions). For our analyses, we reverse-coded those 11 items so that, for all 12 variables, higher values represented higher levels of authoritarian governance. (Following the recoding, all 12 variables were positively inter-correlated; *r*’s ranged from .26 to .77, with median *r* = .54.) We then created a single composite index by standardizing the 12 variables (converting them to z-scores), and computing the mean of these *z*-scores. This 12-item index (Cronbach’s alpha  = .94) served as the primary measure of authoritarian governance.

#### Prevalence of Disease-Causing Parasites

We conducted parallel sets of analyses employing two different measures of parasite prevalence. One measure was developed by Cashdan and Steele [15], based upon the historical prevalence of ten disease-causing pathogens. These authors employed the same source materials employed by Murray and Schaller [29] (described in Study 1 above), and assigned a historical pathogen prevalence score to each of the 186 SCCS societies based upon local conditions (within 200 km) of each society. The specific pathogens coded were leishmanias, trypanosomes, malaria, schistosomes, filariae, dengue, typhus, leprosy, spirochetes, and plague. (Cashdan and Steele generated new codings for 8 of the 10 pathogens; data for the remaining two pathogens—leprosy and spirochetes—were obtained from previously published work [36].) This measure was internally reliable, Cronbach’s alpha  = .81.

The second measure was an index of "total pathogen stress" (SCCS variable #1260) developed previously by Low [36], who drew upon similar source materials but coded fewer categories of parasitic diseases. This index is based on the overall extent to which 7 specific kinds of infectious diseases were present within the region occupied by each society. Across all 186 societies in the SCCS dataset, this 7-item index has Cronbach’s alpha  = .77.

These two indices [15], [36] are highly correlated, *r* = .87 (*p*<.001).

#### Control Variables

Given previous evidence that authoritarianism may be associated with lack of valued resources or by other threats to human welfare [44], [45], [46] we also included 3 additional measures in our analyses.

#### Malnutrition

The SCCS dataset includes 3 variables that assess regular shortages in nutritional resources of the sort that predict chronic malnutrition: ordinary malnutrition, short-term starvation, and seasonal starvation (SCCS variable #'s 1261 – 1263) [47]. Each variable is coded on a 4-point scale. We created a single index by first converting the 3 variables to z-scores, and then computing the mean of these *z*-scores (for the 3-item scale, Cronbach’s alpha  = .63).

#### Famine

The SCCS dataset includes 4 variables that indicate the prevalence of acute famines: occurrence, severity, persistence, and recurrence of famine (SCCS variable #'s 1265, 1267, 1268 and 1269) [47]. Each variable is coded on a 4-point scale. We created a single index assessing threat of famine by first converting the 4 variables to z-scores, and then computing the mean of these *z*-scores (for the 4-item scale, Cronbach’s alpha  = .90). (The malnutrition and famine variables are related, but differ in several important aspects. As defined by these variables, malnutrition is characterized by regular caloric deficiency but is rarely characterized by mortality; in contrast, famine is characterized by significant mortality. Malnutrition is a relatively predictable and chronic state of affairs and so rarely creates alarm within a society; in contrast, famine is an unpredictable and acute event characterized by marked disruptions in community life).

#### Warfare

The SCCS dataset includes separate variables pertaining to the frequency of internal warfare and the frequency of external warfare (SCCS variable #'s 773 and 774) [35]. Each variable is coded on a 4-point scale. We created a single index assessing threat of warfare by first converting these 2 variables to z-scores, and then computing the mean of these *z*-scores (for the 2-item scale, Cronbach’s alpha  = .45.)

### Results and Discussion

Table 2 presents zero-order correlations between variables. Both parasite stress measures were positive predictors of authoritarian governance (*r*'s  = .42 and .29, *p*'s<.01). In addition, the threat of famine also correlated positively with authoritarian governance (*r* = .26, *p* = .01). Neither malnutrition nor warfare was significantly associated with authoritarian governance. (In an additional analysis, we aggregated society-level values within each of the 6 world regions identified by Murdock [48], and thus computed composite measures of pathogen prevalence [15] and authoritarian governance for each of these six culturally-independent world regions. The correlation between these region-level composite variables provides an ancillary test of the parasite stress hypothesis. This correlation was strongly positive, *r*(6)  = .67, *p* = .14.)

**Table 2:** Results from analyses on the Standard Cross Cultural Sample (Study 2): Zero-order correlations between 12-item index of authoritarian governance, two measures of parasite stress, and three measures assessing other threats to health and welfare.

	1	2	3	4	5
1. Authoritarian Governance	—				
2. Pathogen Prevalence (C&S)	.42**	—			
3. Pathogen Stress (L)	.29**	.87**	—		
4. Famine	.26*	–.10	–.06	—	
5. Malnutrition	.05	–.01	–.07	.35**	—
6. Warfare	–.11	–.16	–.30	–.17	–.09

Note: ** *p*<.01, * *p*<.05. "Pathogen prevalence (C&S)" refers to Cashdan & Steele’s [15] index of historical pathogen prevalence; "Pathogen stress (L)" refers to Low's [36] index of total pathogen stress.

We conducted follow-up multiple regression analyses to test whether parasite stress uniquely predicted authoritarian governance, even when controlling for additional threats. In one analysis, the set of predictors included the 3 control variables (famine, malnutrition, and warfare) along with Cashdan and Steele’s [15] measure of historical pathogen prevalence. Results revealed that both pathogen prevalence and the threat of famine were unique predictors of authoritarian governance (*β*'s  = .47 and .36, respectively; *p*'s<.001). In a conceptually identical analysis the predictors included the 3 control variables along with Low's [36] measure of total pathogen stress. The results were inferentially identical: Pathogen stress and the threat of famine were unique predictors of authoritarian governance (*β*'s  = .36 and .34 respectively; *p*'s<.005).

We also performed separate multiple regression analyses on each of the 12 individual variables that comprised the index of authoritarian governance (the 12 variables that Ross [35] identified as indicators of "concentration of political power"). Predictor variables in each analysis included the 3 control variables (famine, malnutrition, and warfare) along with Cashdan and Steele’s [15] measure of historical pathogen prevalence. (Virtually identical results were obtained in separate analyses that instead included Low's [36] measure of pathogen stress.) Results are summarized in Table 3, and reveal that pathogen prevalence uniquely predicted 11 of the 12 variables: Higher levels of pathogen prevalence were associated with ethnographic observations indicating more authoritarian systems of governance. In addition, famine uniquely predicted 8 of the 12 variables, such that greater threat of famine was associated with greater authoritarianism. Malnutrition uniquely predicted 1 of the 12 variables (greater malnutrition was associated with the perception of leaders as *less* powerful). Warfare did not uniquely predict any of these variables.

**Table 3:** Results from analyses on the Standard Cross Cultural Sample (Study 2): Standardized regression coefficients (*β*'s) identifying unique predictive effects of threats due to pathogens, famine, malnutrition and warfare on indicators of authoritarian governance.

SCCS Variable	Pathogens	Famine	Malnutrition	Warfare
#756: Political role differentiation	.33**	.37**	–.21	–.06
#758: Leadership selection basis	.11	.20	–.12	–.11
#759: Perceptions of leader's power	.40**	.44**	–.25*	–.06
#761: Checks on leader's power	.31**	.30*	–.13	–.07
#762: Removal of bad leaders	.33*	.33**	–.02	.05
#763: Leader's exercise of authority	.48**	.24*	–.09	.09
#764: Depth of decision making bodies	.35**	.23*	–.09	.02
#766: Collective decision making	.33**	.16	–.10	.00
#772: Litigation for binding decisions	.46**	.28*	–.16	.05
#776: Formal enforcement of decisions	.43**	.45**	–.06	.08
#777: Prevalence of enforcement specialists	.40**	.17	–.06	.01
#784: Prevalence of taxation	.34**	.22*	–.01	.01

Note: ** *p*<.01, * *p*<.05. All variables were (re-)coded such that higher values indicate greater concentration of political power (i.e., higher levels of authoritarian governance).

Overall, these results support the parasite stress hypothesis, with both the authoritarian composite measure and all but one of its twelve constituent parts producing convergent results. These results also suggest that the prevalence of a disease-irrelevant threat—famine—may also uniquely encourage authoritarian political governance, which is consistent with past research that links other threats to authoritarianism [41], [42].

## General Discussion

Results from both studies provide empirical substantiation for the hypothesis that societal differences in authoritarian governance may result, in part, from ecological variation in the prevalence of disease-causing parasites. Study 1 was designed to address one specific alternative explanation for a previously documented relation between parasite stress and contemporary nation-level markers of authoritarianism [7]. Results revealed that the relation between parasite prevalence and authoritarian governance was mediated by individual authoritarianism—a result that is consistent with the parasite stress hypothesis, and inconsistent with an alternative explanation based solely on the colonial spread of political and economic institutions. (These results do not challenge the important role of colonial history in explaining contemporary nation-level differences in political and economic outcomes [31], [32]. The results simply indicate that a colonial-spread-of-institutions process cannot provide a complete explanation for the relation between parasite prevalence and authoritarianism.)

Study 2 was designed to provide a complementary analysis in a sample of traditional small-scale societies. Results revealed that parasite prevalence predicted the level of authoritarian governance across a diverse sample of 90 small-scale societies within the Standard Cross Cultural Sample (SCCS). These results provide further, empirically independent evidence of the hypothesized relation between parasite stress and authoritarian governance.

The magnitude of the statistical relationships between parasite prevalence and authoritarian governance differed across the two studies: These relations were more modest in the sample of small-scale societies than in the sample of modern geopolitical regions. (Similar differences in magnitude are evident in empirical results linking pathogen stress to collectivist values [8], [15]) Why might this be? One possibility is that, when using contemporary nation states as units of analysis, the relationship is more likely to be spuriously inflated (due, for instance, to conceptually independent processes such as those involved in European colonialism). Another possibility is that, when using the SCCS dataset, the relationship is more likely to be artificially attenuated (due to the measurement error that almost certainly attends any attempt to turn ethnographers' qualitative observations into numerical codings). These considerations suggest caution in drawing any conclusions about actual effect sizes, and attest further to the value of using multiple methods (and multiple samples) to test functional hypotheses of cross-cultural differences.

In addition to parasite prevalence, Study 2 also assessed several additional, conceptually distinct forms of threat to human welfare: malnutrition, famine, and warfare. Results revealed that both parasite prevalence and famine uniquely predicted variability in authoritarian governance. These results suggest that the societal implications of parasite stress (and the societal implications of famine) are distinct from the implications of other variables that might also affect individual fitness and mortality. This conclusion is consistent also with psychological evidence showing that, while other threats can also influence individuals' conformist and ethnocentric attitudes, the perceived threat of infectious disease has effects that are empirically unique and, often, especially powerful [13], [14], [21].

The finding that the threat of famine predicts authoritarian governance in small-scale societies is also convergent with psychological research showing that individual-level authoritarianism is generally higher during times of resource scarcity [42], [49]. Two other threats to human welfare (malnutrition and warfare) had negligible relations with authoritarian governance in small-scale societies. Malnutrition may fail to exact any substantial societal-based influence due to its chronic and enduring nature (whereas famines are acute, and therefore more threat-like). The null result for warfare is perhaps more surprising, given the prevailing belief that authoritarian governments are more likely to go to war [50]. This null result may simply be due to scale. The war-like nature of authoritarian regimes has typically been ascribed to large nation states; the same principles may not apply small-scale societies of the sort represented in the SCCS.

Although these empirical results provide evidence that ecological variation in parasite stress (as well as famine) uniquely predicts societal-level differences in authoritarian governance, these results cannot address deeper questions about the specific underlying processes through which this relation may have emerged. Although the pattern of results is consistent with previous research in the psychological sciences that documents specific cognitive and behavioral changes that occur when individuals perceive that they are vulnerable to infectious diseases, additional mechanisms may also plausibly explain the same societal outcomes [51], [52]. Nor can these kinds of results distinguish whether the unique effects of parasite stress and famine reflect the operation of a single underlying mechanism that responds to any kind of stressor or threat, or whether these two effects reflect the complementary operation of multiple mechanisms that are each functionally attuned to different forms of threat. A simple appeal to parsimony favors the former interpretation. Considerable evidence at an individual level of analysis—including experiments that reveal different effects of different threats on attitudes relevant to authoritarianism—suggests the latter [13], [21], [53].

Although one cannot confidently draw inferences about individual-level processes from population-level data, the results of Study 1 may have other implications at the psychological level of analysis. It has been suggested that an authoritarian personality serves a self-protective function [54]. Consequently, rather than being a stable trait, individuals' authoritarian tendencies may temporarily increase when threats are psychologically salient [39], [55], [56], [57]. Our results provide novel evidence of a relationship between a conceptually distinct form of threat—the threat of infectious disease—and individuals' authoritarian tendencies. This relationship is consistent with a wide range of additional evidence indicating that individuals are sensitive to disease-connoting cues within their immediate environment, and respond to these cues with functionally adaptive shifts in cognition and behavior [58], [59].

These results have further implications for understanding the direction of the presumed causal relation between individual-level authoritarian attitudes and state-level authoritarian governance. Are people who live within authoritarian states more likely to adopt authoritarian attitudes? Or are people who hold authoritarian attitudes more likely to give rise to authoritarian governments? By including an additional variable (parasite prevalence), and using mediation analyses to test the direct and indirect implications of this variable, Study 1 addressed these questions in a novel manner. Results suggest that, consistent with some lines of speculation [56], individual-level authoritarianism shapes political systems, rather than political systems shaping individual attitudes (although, of course, neither causal path necessarily operates at the exclusion of the other).

In addition to their conceptual implications, these results may also have useful implications for predicting the collateral consequences of health-related public policies. If indeed parasite stress has unique causal implications for authoritarian governance, then disease-eradication programs may not only have direct consequences for human health, they may also have indirect consequences for individual rights, civil liberties, and political freedoms. (Thornhill and colleagues [7] noted that the democratic transitions in North America and Europe were preceded by dramatic reductions in the prevalence of infectious disease.) There may also be implications for reduced levels of xenophobia and other prejudices that are linked to authoritarian attitudes [1], [2], [60], [61], and for increased levels of creativity, innovation, and open-mindedness more generally.
